# Humoral immune responses to inactivated COVID-19 vaccine up to 1 year in children with chronic hepatitis B infection

**DOI:** 10.3389/fcimb.2023.1201101

**Published:** 2023-06-29

**Authors:** Yingzhi Zhou, Zhiwei Chen, Yi He, Xiaorong Peng, Yunan Chang, Aoxue Tan, Hu Li, Dachuan Cai, Peng Hu, Min Chen, Mingli Peng, Hongmei Xu, Hong Ren

**Affiliations:** ^1^ Department of Infectious Diseases, Children’s Hospital of Chongqing Medical University, National Clinical Research Center for Child Health and Disorders, Ministry of Education Key Laboratory of Child Development and Disorders, Chongqing Key Laboratory of Child Infection and Immunity, Chongqing, China; ^2^ Department of Infectious Diseases, Key Laboratory of Molecular Biology for Infectious Diseases, Ministry of Education, Institute for Viral Hepatitis, the Second Affiliated Hospital, Chongqing Medical University, Chongqing, China

**Keywords:** adolescent, chronic hepatitis B, COVID-19, omicron, inactivated vaccine, antibody response, memory B cells

## Abstract

**Background:**

Inactivated SARS-CoV-2 vaccination has recently been approved for children aged 3-17 years in China. However, data on long-term humoral responses to inactivated vaccines in children with chronic hepatitis B (CHB) are still limited.

**Methods:**

In this prospective observational study, CHB children after primary inactivated SARS-CoV-2 vaccines were recruited consecutively and followed up for 1 year. CHB adults from another cohort study (NCT05007665) were used as a control. The receptor-binding domain IgG antibody (anti-RBD-IgG), neutralizing antibody (NAb), neutralization against Omicron (BA2.12.1, BA.4 and BA.5), and memory B -cell (MBC) responses were evaluated.

**Results:**

Overall, 115 CHB children and 351 CHB adults were included in this analysis. The antibody titers decreased over the first ~180 days and then plateaued up to 1 year in CHB children. However, lower and faster declines in antibody responses were observed in CHB adults. Interestingly, the seroprevalence of antibodies was still high after over 8 months in CHB children (anti-RBD-IgG [90%] and NAbs [83%]). However, neutralization against Omicron subvariants was significantly reduced in CHB children (-3.68-fold to -8.60-fold). Notably, neutralization against the BA.5 subvariant was obviously diminished in CHB children compared with adults. Moreover, CHB children had similar RBD-specific MBCs but higher RBD-specific atypical MBCs compared with adults.

**Conclusion:**

Inactivated vaccination could elicit more robust and durable antibody responses to the wild-type SARS-CoV-2 strain in CHB children than in CHB adults but showed inferior responses to Omicron subvariants (especially to the BA.5 strain). Hence, new Omicron-related or all-in-one vaccines are needed immediately for CHB children.

## Introduction

Ongoing coronavirus disease 2019 (COVID-19), caused by severe acute respiratory syndrome coronavirus 2 (SARS-CoV-2), has been pandemic worldwide for over three years (World Health Organization COVID-19 Dashboard). Due to the continuing evolution of SARS-CoV-2, Omicron has become the predominant lineage to date (Wang et al., 2022). Unfortunately, children showed a higher vulnerability and worse outcome during Omicron infection than during wild-type infection (Khemiri et al., 2022).

Vaccination is currently the most effective means to constrain the COVID-19 pandemic (Dagan et al., 2021). Inactivated SARS-CoV-2 vaccines (BBIBP-CorV/CoronaVac) have recently been approved for children aged 3-17 years in China (Xue and Shen, 2021). Several studies have shown that vaccines against COVID-19 are safe and effective for the general child population (Creech et al., 2022; Dowell et al., 2022; Klein et al., 2022; Vadrevu et al., 2022). Considering that the presence of comorbidities was associated with worse outcomes among children with Omicron infection (Butt et al., 2022; Shen et al., 2023), more attention should be given to this special population. However, the safety and immunogenicity of vaccines in children with comorbidities are limited.

Patients with liver diseases are highly vulnerable to COVID-19 (Premkumar and Kedarisetty, 2021), and hepatitis B virus infection is the most common etiology in China (Wang et al., 2014). Our previous study showed that adult patients with chronic hepatitis B virus infection (CHB) elicited an inferior antibody response compared with healthy controls (HCs) in the early stage after inactivated vaccination (He et al., 2022). In addition, memory B cells (MBCs) are important for maintaining long-term immune memory function (Ogega et al., 2021). Our previous study showed that MBC responses did not change over time after vaccination in adult patients with CHB (He et al., 2022). However, the safety and humoral immune responses of inactivated SARS-CoV-2 vaccination in CHB children are still unclear.

In this prospective observational study, we recruited 115 CHB children, followed up for one year, and evaluated the safety, antibody responses (especially to Omicron) and MBC responses after primary inactivated SARS-CoV-2 vaccination.

## Participants and methods

### Study design and participants

In this observational study, CHB children (age < 18 years) who completed two doses (primary) of inactivated SARS-CoV-2 vaccines (BBIBP-CorV/CoronaVac, 0.5ml per dose) were consecutively recruited at the Children’s Hospital of Chongqing Medical University beginning on September 14, 2021. The inclusion criterion for CHB patients was hepatitis B surface antigen positivity for more than 6 months. For all participants, the following conditions were excluded: a) history of SARS-CoV-2 infection; b) coinfection with human immunodeficiency virus or hepatitis C virus; c) autoimmune disease; d) liver cirrhosis or malignant tumor; e) use of immunosuppressants; f) pregnancy; and g) diabetes or hypertension. In addition, after matching several potential confounding factors, such as sex, vaccine types and antiviral situation, 351 CHB adults who had received two doses of inactivated vaccines (BBIBP-CorV/CoronaVac, 0.5ml per dose) from a previous study cohort (NCT05007665) were used as the control group; they were recruited at the Second Affiliated Hospital of Chongqing Medical University (He et al., 2022).

This study was approved by the Ethics Committee of Children’s Hospital of Chongqing Medical University and the Second Affiliated Hospital of Chongqing Medical University and registered at www.chictr.org.cn (ChiCTR2100050267). This study conformed to the ethical guidelines of the Declaration of Helsinki. Written informed consent was obtained from all participants and their guardians.

### Data and sample collection

The participants’ demographic characteristics, medical history, and clinical data were obtained from questionnaires or electronic medical records. Adverse events (AEs) of vaccination were collected by oral questionnaire. At each visit time, sera were used to detect the antibody responses, and peripheral blood mononuclear cells were used to evaluate SARS-CoV-2-specific MBC responses.

### Chemiluminescence immunoassays

The anti-receptor-binding-domain IgG (anti-RBD-IgG) and neutralizing antibodies (NAbs) in serum samples were detected using capture chemiluminescence immunoassays by MAGLUMI X8 (Snibe, Shenzhen, China), as described in our previous study (Chen et al., 2023). The sensitivity and specificity of anti-RBD IgG were 100% and 99.6%, respectively, and those of NAbs tests were both 100%. The cutoff values were 1 AU/mL for anti-RBD-IgG and 0.15 μg/mL for NAbs.

### Pseudotyped viral neutralization assay

Serially diluted serum samples or controls were incubated with diluted pseudotyped HIV-1 viruses expressing the spike of SARS-CoV-2 wild type (WT) and Omicron subvariants (BA.2.12.1, BA.4 and BA.5) and then added to confluent 293T-ACE2 cell monolayers. After incubation, the luciferase value (relative light unit) of all samples was detected by a luminometer. Serum samples were diluted with twofold serial dilutions starting from 1:10. The 50% pseudovirus neutralization titers (pVNT50) were calculated using the Reed-Muench method. The detailed steps are illustrated in the **Supplementary Methods**.

### RBD-specific memory B cells

The SARS-CoV-2-specific MBCs were examined by flow cytometry, as described in a previous study (Chen et al., 2023). In brief, biotinylated SARS-CoV-2 spike RBD protein (Sino Biological, 40592-V08H2-B) was mixed with streptavidin BV421 (Biolegend, 405225) at a 4:1 molar ratio for 1 hour at 4 °C to obtain the antigen probe. Freshly drawn peripheral blood from participants was processed and stained with specific fluorochrome-conjugated monoclonal antibodies (mAbs), including an antigen probe (1:33.3), anti-CD3-PerCP5.5 (300430, Biolegend, 1:50), anti-CD19-APC (302212, Biolegend, 1:50), anti-CD27-PE (356406, Biolegend, 1:50), and anti-CD21-Alexa Fluor^®^ 700 (354918, Biolegend, 1:50), to perform flow cytometric analysis. Multiparameter flow cytometry detection was performed by CytoFLEX (Beckman Coulter, USA), and the data were analyzed using FlowJo software (10.0.7r2, Treestar).

### Statistical analysis

SPSS 27.0 and GraphPad Prism 8.0.2 were used to analyze the data. The chi-square test was used for categorical variables. For continuous variables, the Mann-Whitney U test or Kruskal-Wallis test was used for comparisons between two or more groups. The results were corrected for multiple comparisons using the Bonferroni test. The Spearman method was used to analyze correlations between variables. Modeling for the best fit curve (one phase decay versus simple linear regression) was performed to present the kinetic changes in humoral immune responses over time. The best curve fit was defined by an extra sum-of-squares F Test, selecting the simpler model unless P < 0.05. Univariate and multivariate binary logistic regression analyses were performed to investigate factors affecting antibody responses to inactivated vaccines in CHB children. A P value < 0.05 (two-tailed) was considered statistically significant.

## Results

### Demographic and clinical characteristics of the participants

From September 14, 2021 to September 14, 2022, a total of 115 CHB children were enrolled in this analysis. The demographic and clinical characteristics of CHB children are shown in **Table 1**, and those of the 351 CHB adults (control group) are shown in **Supplementary Table 1**. The median age of CHB children was 9 years (IQR 6-12). Over half of the CHB children were male (54.8% [63/115]). A total of 36.5% (42/115) of CHB children were HBeAg negative, and 53.9% (62/115) of them were receiving antiviral therapy. The proportion of patients with HBV DNA < 2000 IU/ml was 48.7% (56/115) (**Table 1**).

**Table 1:** The demographic and clinical characteristics of CHB children.

Variables	CHB children (n=115)
Age (years)	9 (6-12)
Sex (male, n (%))	63 (54.8%)
BMI (kg/m^2)	16.02 (14.51-18.30)
Vaccine type^#^	
BBIBP-CorV, n (%)	38 (33.0%)
CoronaVac, n (%)	58 (50.4%)
Mixed vaccination, n (%)	15 (12.7%)
WBC (10^9/L)	6.10 (5.24-7.42)
PLT (10^9/L)	262.5 (223.3-299.8)
ALT (U/L)	26 (19-41)
Normal ALT, n (%)	86 (74.8%)
HBeAg negative, n (%)	42 (36.5%)
HBV DNA ≤ 2000 IU/ml, n (%)	56 (48.7%)
Antiviral treatment (Yes, n (%))	62 (53.9%)
Days after two doses of vaccines (n=205)	141.0 (65.5-221.0)
Blood collection frequency	
Multiple time point donors (two to five times)	50.4% (58/115)
Single–time point donors	49.6% (57/115)

CHB, chronic hepatitis B; IQR, interquartile range; BMI, body mass index; WBC, white blood cell; PLT, platelet; ALT, alanine aminotransferase; HBeAg, hepatitis B e antigen. ^#^ Vaccine types of 4 children are unclear.

Overall, 205 blood samples from the 115 CHB children were collected, and of them, 58 patients provided longitudinal blood samples during the observation period (two to five time points; **Table 1**). The median elapsed time between primary vaccination and blood sampling in CHB children was 39.0 days (IQR 30.5-47.0) at T1, 92.0 days (72.0-107.0) at T2, 142.0 days (128.5-161.0) at T3, 213.5 days (204.0-229.5) at T4, and 279.0 days (254.0-318.0) at T5. A total of 478 samples from 351 CHB adults were collected, and the blood sampling was 33.0 days (IQR 29.0-43.0) at T1, 97.0 days (77.5-101.8) at T2, 174.0 days (171.0-175.0) at T3, and 191.0 days (186.0-194.0) at T4 (**Supplementary Table 1**).

### Antibody responses to inactivated SARS-CoV-2 vaccine

**Figure 1:**
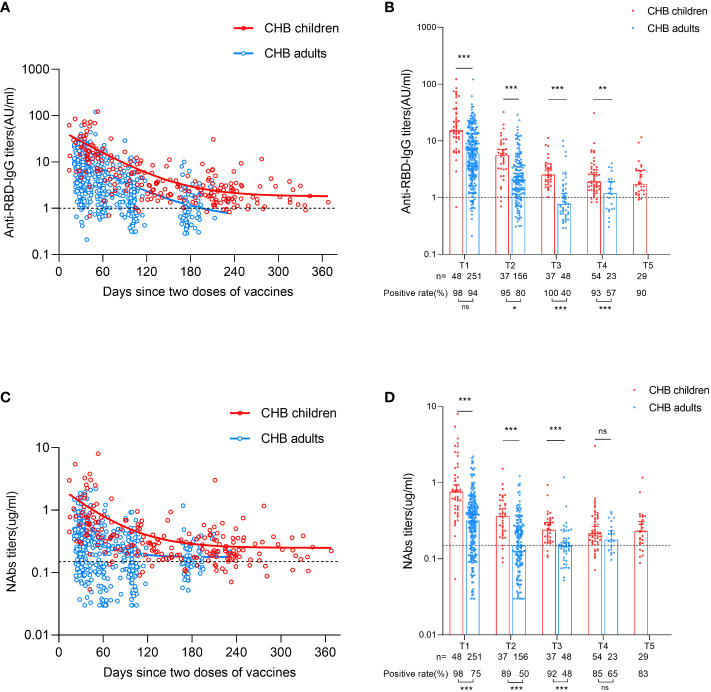
Comparisons of antibody responses after primary inactivated vaccination in CHB children and adults. **(A)** The kinetic changes in the titers of anti-RBD-IgG over time in CHB children (n=205) and adults (n=478). **(B)** The seropositivity rates and titers of anti-RBD-IgG at T1, T2, T3, T4 and T5 in CHB children and adults. **(C)** The kinetic changes in the titers of NAbs over time in CHB children and adults. **(D)** The seropositivity rates and titers of NAbs at T1, T2, T3, T4 and T5 in CHB children and adults. The plots were shown as median values with the 95% confidence interval. The dotted lines represent cut-off values. *P < 0.05; **P < 0.01; ***P < 0.001; ns, not statistically significant. anti-RBD-IgG, anti-receptor-binding-domain immunoglobulin G; NAbs, neutralizing antibodies. The Mann-Whitney U test was used for comparisons between groups. Modeling for the best fit curve (one phase decay) was performed to present the kinetic changes in antiboy responses over time.

First, we depicted the dynamic changes in the antibody response after primary inactivated vaccination in CHB children. As shown in **Figure 1A** and **Supplementary Figure 1A**, the titer of anti-RBD-IgG decreased over the first ~180 days and then plateaued up to 1 year in CHB children after primary vaccination. Compared with CHB children, a lower and faster decline in the anti-RBD-IgG response was observed in CHB adults after vaccination (anti-RBD-IgG seropositivity rates: T1: 98% vs. 94%, *P*=0.239; T2: 95% vs. 80%, *P*=0.036; T3: 100% vs. 40%, *P*=0.000; T4: 93% vs. 57%, *P*=0.000; titer (AU/ml): T1: 15.210 vs. 5.960, *P*=0.000; T2: 5.548 vs. 2.049, *P*=0.000; T3: 2.527 vs. 0.773, *P*=0.000; T4: 1.908 vs. 1.191, *P*=0.003). For CHB children, the positive rate of anti-RBD-IgG titer was still high (90%) at T5 (over 8 months) after vaccination (**Figure 1B**). Similar results were also observed in NAbs responses (NAbs seropositivity rates: T1: 98% vs. 75%, *P*=0.000; T2: 89% vs. 50%, *P*=0.000; T3: 92% vs. 48%, *P*=0.000; T4: 85% vs. 65%, *P*=0.067; titer (µg/ml): T1: 0.759 vs. 0.316, *P*=0.000; T2: 0.360 vs. 0.150, *P*=0.000; T3: 0.240 vs. 0.148, *P*=0.000; T4: 0.215 vs. 0.178, *P*=0.115; **Figures 1C, D**, **Supplementary Figure 1C**).

**Figure 2:**
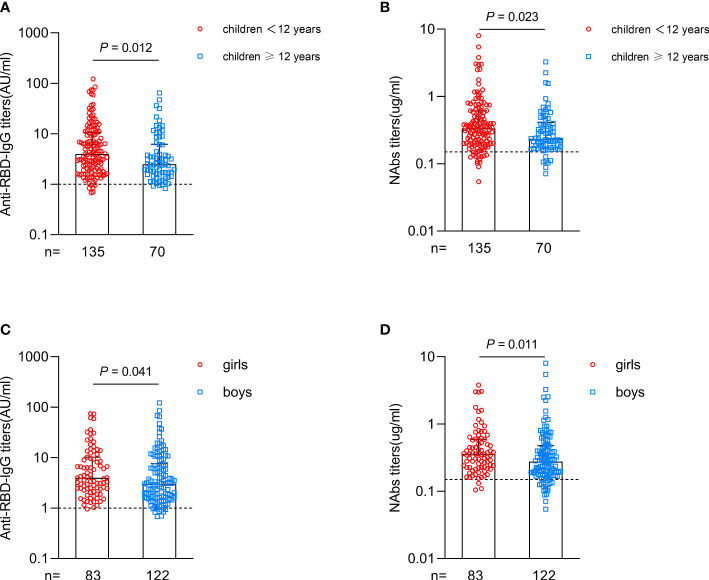
Antibody responses after primary inactivated vaccination in different subgroups of CHB children. **(A, B)** Comparisons of the titers of anti-RBD-IgG **(A)** or NAbs **(B)** in CHB children grouped at 12 years of age. **(C, D)** Comparisons of the titers of anti-RBD-IgG **(C)** or NAbs **(D)** in CHB children grouped by sex. The plots were shown as median values with the 95% confidence interval. The dotted lines represent cut-off values. anti-RBD-IgG, anti-receptor-binding-domain immunoglobulin G; NAbs, neutralizing antibodies. The Mann-Whitney U test was used for comparisons between groups.

Interestingly, subgroup analysis in CHB children showed that the antibody responses were higher in younger children (age < 12 years) than in older children after vaccination (anti-RBD-IgG: 3.981 vs. 2.487, *P*=0.012; NAbs: 0.335 vs. 0.235, *P*=0.023; **Figures 2A, B**). Girls had higher antibody titers than boys after vaccination (anti-RBD-IgG: 3.976 vs. 3.053, *P*=0.041; NAbs: 0.356 vs. 0.276, *P*=0.011; **Figure 2C, D**). Further subgroup analysis of the levels of ALT and HBV DNA, HBeAg status, antiviral treatment and vaccine type in CHB children revealed no significant difference in antibody responses between different subgroups (**Supplementary Figure 2**).

Altogether, primary inactivated SARS-CoV-2 vaccination could elicit more robust and durable antibody responses in CHB children than in CHB adults.

### Neutralization against Omicron variants to inactivated SARS-CoV-2 vaccine

**Figure 3:**
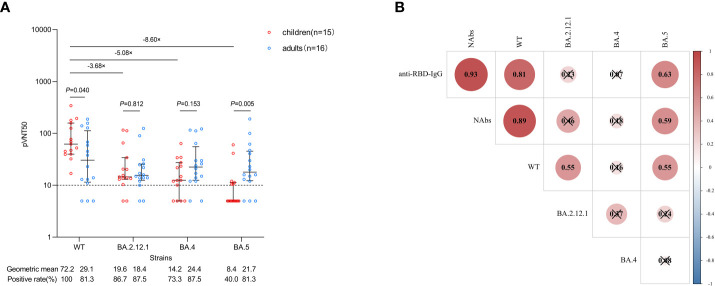
Neutralization against WT strain and Omicron subvariants after primary inactivated vaccination. **(A)** Comparisons of the neutralization against WT strain and Omicron subvariants in CHB children (n=15) and CHB adults (n=16). **(B)** Correlations between anti-RBD-IgG, NAbs and the neutralization. The plots were shown as geometric mean values with the 95% confidence interval. The dotted lines represent detection limit. The values of less than 10 for the pVNT50 indicate negative samples and were counted as 5. Red indicates a positive correlation between two variables, blue indicates a negative correlation, the number in each circle indicates the correlation coefficient, and the symbol “×” indicates not statistically significant. anti-RBD-IgG, anti-receptor-binding-domain immunoglobulin G; NAbs, neutralizing antibodies; WT, wild type; pVNT50, 50% pseudovirus neutralization titers.The Mann-Whitney U test was used for comparisons between groups. The Spearman method was used to analyze correlations between variables.

Next, we focused on neutralization against the WT strain and three Omicron dominant subvariants (BA.2.12.1, BA.4 and BA.5) by pseudovirus neutralization assay. Serum from 15 CHB children and 16 CHB adults at 1 month after primary vaccination was used for analysis, and the characteristics of the participants are shown in **Supplementary Table 2**. Compared with WT, neutralization against BA.2.12.1, BA.4 and BA.5 subvariants was obviously reduced in CHB children (-3.68-fold, -5.08-fold, and -8.60-fold, respectively, all P<0.05; **Figure 3A**). Consistent with the trend of anti-RBD-IgG and NAbs, the neutralization titer against WT in CHB children was also higher than that in CHB adults (72.2 vs. 29.1, *P*=0.040). However, the neutralization titers against BA2.12.1 and BA.4 between the two groups were similar (both *P >*0.05). Notably, the neutralization titers against BA.5 in CHB children were significantly lower than in CHB adults (8.4 vs. 21.7, *P*=0.005) (**Figure 3A**).

We also analyzed the correlations between anti-RBD-IgG, NAbs and neutralization. As shown in **Figure 3B**, there were strong correlations between the titers of anti-RBD-IgG and NAbs and the neutralization titer against WT (*P*<0.05). The titers of anti-RBD-IgG and NAbs were moderately correlated with the neutralization titer against BA.5 (*P*<0.05). However, the correlation of the titers of anti-RBD-IgG and NAbs with the neutralization titer against BA.2.12.1 or BA.4 was not observed (*P*>0.05). In addition, the neutralization titer against WT was moderately correlated with BA.2.12.1 and BA.5 (*P*<0.05), but no correlation was observed between the three Omicron subvariants (*P*>0.05).

In general, neutralization against Omicron subvariants was inferior in CHB children than in CHB adults, especially for the BA.5 subvariant.

### RBD-specific MBC responses to inactivated SARS-CoV-2 vaccine

**Figure 4:**
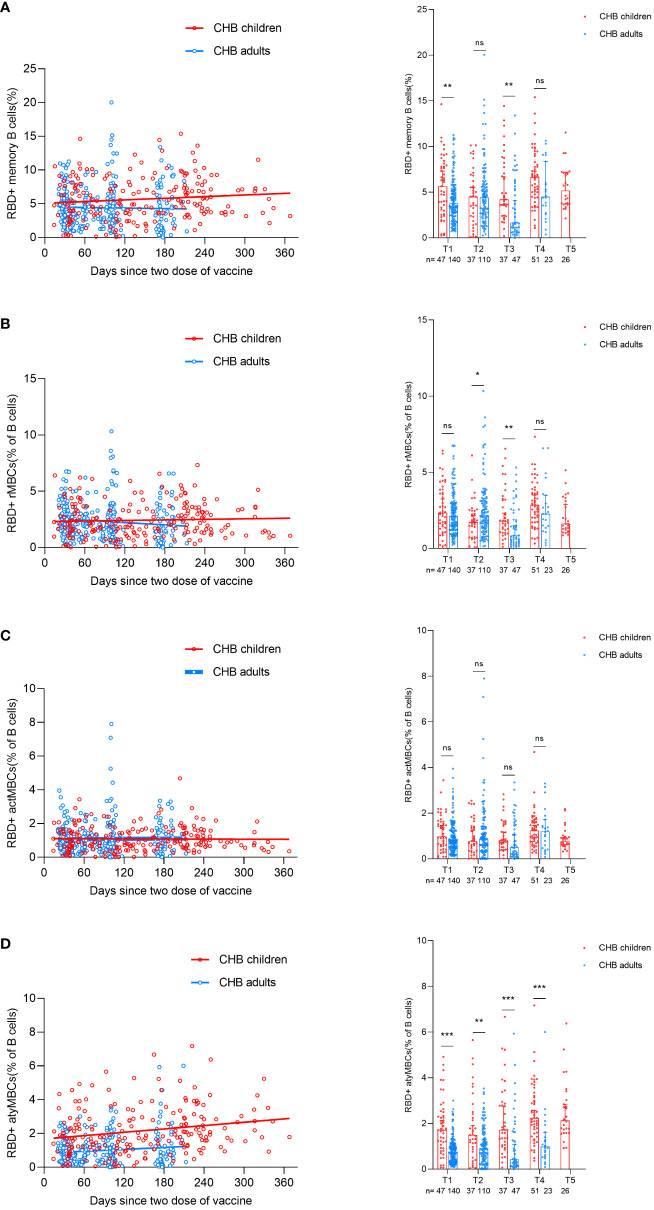
Comparisons of RBD-specific memory B-cell responses after primary inactivated vaccination in CHB children and adults. **(A–D)** The kinetic changes in the frequencies of RBD-specific MBCs **(A)** and the three subtypes of RBD-specific MBCs: RBD-specific rMBCs **(B)**, RBD-specific actMBCs **(C)**, and RBD-specific atyMBCs **(D)** over time in CHB children(n=198) and adults(n=320). The plots were shown as median values with the 95% confidence interval. *P < 0.05; **P < 0.01; ***P < 0.001; ns, not statistically significant. RBD, receptor-binding-domain; MBCs, memory B cells; rMBCs, resting MBCs; actMBCs, activate MBCs; atyMBCs, atypical MBCs. The Mann-Whitney U test was used for comparisons between groups. Modeling for the best fit curve (simple linear regression) was performed to present the kinetic changes in antibody responses over time.

After we evaluated the antibody responses in CHB children after inactivated vaccination, we next wanted to describe the MBC responses in CHB children after vaccination. The gating strategy of flow cytometry for the target cell population is shown in **Supplementary Figure 3**. Overall, the frequencies of RBD-specific MBCs (percentage of total B cells) in CHB children and CHB adults were relatively stable over time after vaccination (**Figure 4A**). In total, the frequencies of RBD-specific MBCs in CHB children were significantly higher than those in CHB adults (T1: 5.64% vs. 3.77%, *P*=0.008; T2: 4.51% vs. 4.43%, *P*=0.830; T3: 4.21% vs. 1.67%, *P*=0.002; T4: 6.67% vs. 4.52%, *P*=0.056; **Figure 4A**).

Further subgroup analysis of three subsets of MBCs showed that the frequencies of classical MBCs (resting MBCs (rMBCs) and activated MBCs (actMBCs)) in CHB children were similar to those in CHB adults (rMBCs: T1: 2.35% vs. 2.12%, *P*=0.393; T2: 1.73% vs. 2.14%, *P*=0.023; T3: 1.79% vs. 0.84%, *P*=0.007; T4: 2.87% vs. 2.27%, *P*=0.142; actMBCs: T1: 0.98% vs. 0.80%, *P*=0.162; T2: 0.78% vs. 0.89%, *P*=0.388; T3: 0.81% vs. 0.51%, *P*=0.084; T4: 0.75% vs. 0.46%, *P*=0.787; **Figures 4B, C**). Interestingly, CHB children had significantly higher frequencies of atypical MBCs (atyMBCs) than CHB adults at each visit timepoint (T1: 1.72% vs. 0.76%, *P*=0.000; T2: 1.50% vs. 0.92%, *P*=0.004; T3: 1.72% vs. 0.44%, *P*=0.000; T4: 2.24% vs. 0.99%, *P*=0.000; **Figure 4D**).

### Risk factors related to antibody responses after primary inactivated vaccination in CHB children

**Figure 5:**
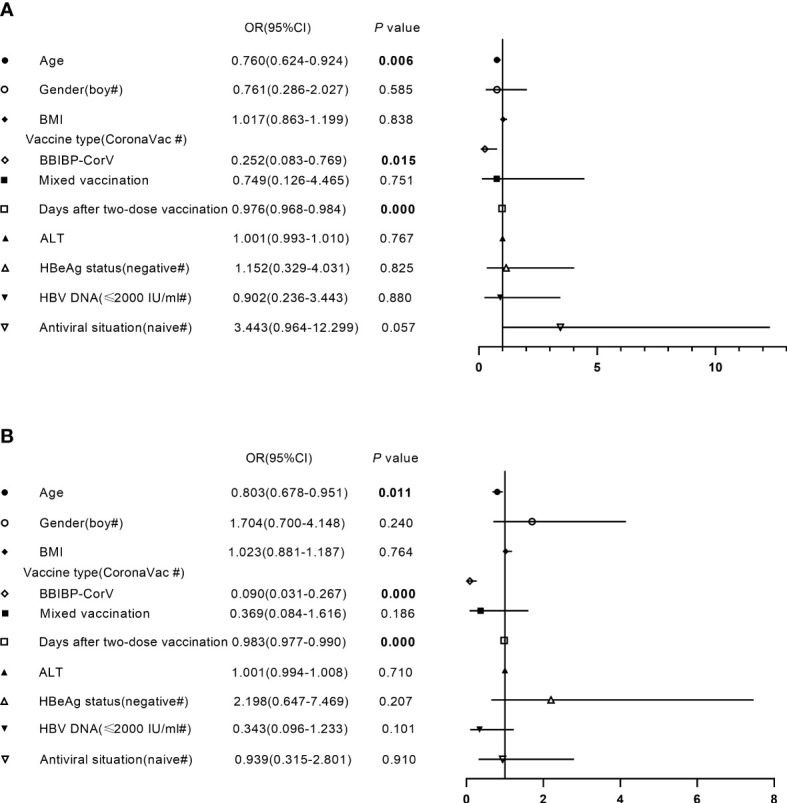
Factors independently associated with antibody level after primary inactivated vaccination in CHB children. **(A, B)** Factors independently associated with the titers of anti-receptor-binding-domain immunoglobulin G **(A)** or neutralizing antibodies **(B)** after primary inactivated vaccination in CHB children. BMI, body mass index; ALT, alanine aminotransferase; HBeAg, hepatitis B e antigen; OR, odds ratio; CI, confidence interval. Multivariate binary logistic regression analysis was performed to investigate factors affecting antibody responses to inactivated vaccines in CHB children.

To better understand the factors affecting the antibody response to the inactivated SARS-CoV-2 vaccine, CHB children were divided into two groups based on the median titers of anti-RBD-IgG (3.259 AU/ml) and NAbs (0.300 µg/ml). Bivariate associations between demographic and clinical characteristics and antibody levels are summarized in **Supplementary Table 3**. As shown in **Figure 5**, younger age, vaccination with CoronaVac, and shorter postvaccination period were significantly associated with higher antibody levels after inactivated vaccination in CHB children, while other parameters such as sex, BMI, the levels of ALT and HBV DNA, HBeAg status, and antiviral situation were not associated with antibody level after adjustment.

### Safety of COVID-19 vaccination in CHB children

Most children or their guardians reported short-term local pain and swelling after vaccination. No serious adverse events or HBV reactivation were observed in CHB children.

## Discussion

To date, there is still a data gap in the long-term humoral responses to SARS-CoV-2 vaccines in CHB children. In this study, we evaluated the humoral responses up to 1 year after primary inactivated vaccination in CHB children. Our results showed that CHB children had robust and durable responses to WT binding antibodies compared with CHB adults but inferior responses to Omicron subvariants (especially for BA.5 strain) and immune memory.

Inactivated SARS-CoV-2 vaccination has recently been recommended for children in China (Xue and Shen, 2021). However, the long-term dynamic changes in humoral responses in CHB children after vaccination are still unknown. In this study, we found that the antibody responses in CHB children after vaccination were more robust than those in CHB adults, which was similar to studies on other COVID-19 vaccines (Gupta et al., 2022; Rosa Duque et al., 2022). Higher antibody responses were observed in younger CHB children after vaccination, and this phenomenon also occurred in other previous studies (Creech et al., 2022; Vadrevu et al., 2022). Interestingly, the high seroprevalences of antibodies were maintained in CHB children during up to 1 year of follow-up, which was different from CHB adults. Several studies suggest that the naïve immune response in children may allow the immune system to evolve more easily to pathogens, making it poised to generate broader immunity to new viruses (Goronzy and Weyand, 2005; Vatti et al., 2017). Hence, CHB children may acquire long-term protection against the WT SARS-CoV-2 strain from vaccination.

With the continual evolution of the SARS-CoV-2 strain, Omicron and its subvariants have become the dominant lineage of SARS-CoV-2 worldwide (Wang et al., 2022). Similar to previous studies, neutralization against Omicron subvariants (BA.2.12.1, BA.4 and BA.5) was also significantly compromised in CHB children, which showed the strong immune escape of Omicron (Liu et al., 2021). This may be the reason for the increasing SARS-CoV-2 infection and hospitalization rates of children during the Omicron predominance (Shen et al., 2023). Notably, inconsistent with the higher antibody responses against WT in CHB children, an obvious decrease in the neutralization against the BA.5 subvariant in CHB children compared with CHB adults was observed, which was similar in healthy children who received other COVID-19 vaccines (Gupta et al., 2022). This indicated that children’s immune systems may be more specialized than those of adults; that is, the capability of cross-reactivity to the Omicron variant is inferior in children after receiving WT SARS-CoV-2 vaccines. Therefore, Omicron-related or all-in-one COVID-19 vaccines are urgently well designed and developed and vaccinated for this special population.

Preexisting protective antibodies act as a first line of defense against reinfection, and memory B cells produced after viral infection or vaccination are considered a second line of defense against reinfection (Inoue et al., 2018). In this study, the frequencies of RBD-specific MBCs in CHB children were relatively stable up to 1 year after vaccination. In total, the frequencies of classical RBD-specific MBCs were similar in CHB children and adults. However, CHB children had higher RBD+atyMBCs, which are part of an alternative lineage of B cells (Sutton et al., 2021). Previous studies have reported that atyMBCs expand in several autoimmune diseases and infectious diseases, and may actively contribute to humoral immune responses and antibody secretion (Wildner et al., 2021; Hopp et al., 2022; Molinos-Albert et al., 2022). AtyMBCs were identified as precursors of autoantibody producing plasma cells in Systemic Lupus Erythematosus(SLE). (Jenks et al., 2018) It has also been reported that atyMBCs may be responsible for secreting autoimmune antibodies that target proteins on the uninfected erythrocytes’membrane, leading to anemia (Rivera-Correa et al., 2019). Besides, atyMBCs are also involved in response to vaccination in humans (Sutton et al., 2021). CD19+IgD+CD27- B cells were reported to be predictors of humoral response to COVID-19 mRNA vaccination in immunocompromised patients (Schulz et al., 2021). Compared to older adults with impaired response to influenza vaccination, the preferential expansion of a population of HA-binding atyMBCs was found in younger adults (Burton et al., 2022). In summary, we have reported an expansion of vaccine-specific atyMBCs following inactivated SARS-CoV-2 immunization in CHB children than in adult patients. The routes to acquire an atypical memory phenotype have not been completely understood till now. Understanding memory B cell formation and recall in relation to age is important for understanding how we can use vaccination to provide effective and durable protection against infection in children, a population seemed to with immature immune and inadequate immune response.

In addition, we evaluated the safety of inactivated vaccination in CHB children, and no serious adverse events or HBV reactivation were observed. Furthermore, we found that only younger age, vaccination with CoronaVac, and shorter postvaccination period were significantly associated with higher antibody level in CHB children, while other parameters such as sex, BMI, level of ALT and HBV DNA, HBeAg status, and antiviral situation were not associated with antibody level after adjustment. This finding is consistent with our previous study in an adult CHB cohort (He et al., 2022).

This study has several limitations. First, the follow-up of CHB children in this study was not good, and only 50.4% of patients provided two more blood samples. Due to the sporadic outbreaks of COVID-19 in China during the observation period, patients are difficult to visit the hospital in a timely manner. Second, the correlation between low neutralization titers against Omicron subvariants and susceptibility to SARS-CoV-2 infection or reinfection in CHB children is still unclear, and more large population studies are needed. Third, T-cell responses were not evaluated in this study, and more data, such as the function of T cells, follicular helper T cells and regulatory T cells, should be evaluated to depict the detailed immune dynamic atlas in CHB children after inactivated vaccination in the future.

In conclusion, our data revealed that inactivated SARS-CoV-2 vaccines were well tolerated and could elicit more robust and durable antibody responses to the WT strain in CHB children than in CHB adults. However, neutralization against Omicron subvariants (especially for the BA.5 strain) was obviously diminished. Therefore, well-designed Omicron-related or all-in-one COVID-19 vaccines and optimized booster vaccination strategies are urgently needed for CHB children.

## Data availability statement

The raw data supporting the conclusions of this article will be made available by the authors, without undue reservation.

## Ethics statement

This study was approved by the Ethics Committee of Children’s Hospital of Chongqing Medical University and the Second Affiliated Hospital of Chongqing Medical University and registered at www.chictr.org.cn (ChiCTR2100050267). This study conformed to the ethical guidelines of the Declaration of Helsinki. Written informed consent was obtained from all participants and their guardians.

## Author contributions

Concept and design: HR, HX, MP. Funding acquisition: HR, HX, MP. Participant recruitment: YZ, ZC, YH, XP, YC, AT. Experiment execution: YZ, YH, MC, MP. Acquisition, analysis or interpretation of data: YZ, ZC, YH, HL, DC, PH, MC, MP, HX, HR. Drafting and critical revision of manuscript: YZ, ZC, YH, MP, HX, HR. All authors contributed to the article and approved the submitted version.
