# Supplementary material for: Humoral immune responses to inactivated COVID-19 vaccine up to 1 year in children with chronic hepatitis B infection
Source: Front Cell Infect Microbiol. 2023 Jun 29;13:1201101. doi: 10.3389/fcimb.2023.1201101 (PMC10339386; doi:10.3389/fcimb.2023.1201101)
Supplement: Supplementary file 1 [file DataSheet_1.docx]

**Supplementary Files**

**Humoral immune responses to inactivated COVID-19 vaccine up to 1 year in children with chronic hepatitis B infection**

Yingzhi Zhou^1†^, Zhiwei Chen^2†^, Yi He^1†^, Xiaorong Peng^1^, Yunan Chang^1^, Aoxue Tan^1^, Hu Li^2^, Dachuan Cai^2^, Peng Hu^2^, Min Chen^2^, Mingli Peng^2*^, Hongmei Xu^1*^, Hong Ren^2*^

Supplementary Methods....................................................................................2

Supplementary Figure 1....................................................................................4

Supplementary Figure 2....................................................................................5

Supplementary Figure 3....................................................................................6

Supplementary Table 1.....................................................................................7

Supplementary Table 2.....................................................................................8

Supplementary Table 3.....................................................................................9

**Supplementary Methods**

**Pseudotyped viral neutralization assay**

Pseudotyped HIV-1 viruses (Sino Biological, Beijing, China) expressing the spike of syndrome coronavirus 2 (SARS-CoV-2) wild type (WT) (Wuhan-1 reference strain) and Omicron subvariants (BA.2.12.1, BA.4 and BA.5) included the spike sequences with the following amino acid changes relative to Wuhan-1:

BA.2.12.1: T19I, Δ24-26, A27S, G142D, V213G, G339D, S371F, S373P, S375F, T376A, D405N, R408S, K417N, N440K, L452Q, S477N, T478K, E484A, Q493R, Q498R, N501Y, Y505H, D614G, H655Y, N679K, P681H, S704L, N764K, D796Y, Q954H, N969K.

BA.4/5: As for BA.2 with additional Δ69-70, L452R, and F486V mutations, but lacking L452Q, Q493R, and S704L mutations.

Pseudovirus neutralization titers were detected as follows: 293T-ACE2 cells (OEC001, Sino Biological) were seeded in 96-well plates (3×10^4^ cells/well) in the dulbecco’s modified eagle medium supplemented with 10% fetal bovine serum (FBS) to produce a monolayer at the time of infection. Serially diluted samples or controls were incubated with pseudovirus at 37 ℃ for 1 hour and then added to confluent 293T-ACE2 monolayers in 96-well plates. The plates were then cultured in a 5% CO_2_ incubator at 37 ℃ for 70 hours. After incubation, the luciferase value [relative light unit (RLU)] of all samples was detected by the luminometer (LB9630, Berthold Technologies, Baden-Württemberg, Germany). Serum samples were diluted with two-folded serial dilution starting from 1:10. For each experiment, positive controls (pseudovirus only), negative controls (sera from individuals without a history of SARS-CoV-2 infection or vaccination) and antibody controls (antibodies with neutralizing activity) were set up at the same time. Each measurement was performed in duplicate. Inhibition rate (%) was calculated as “1 - (average RLU of sample - average RLU of negative control) ÷ (average RLU of positive control - RLU of negative control)”. The 50% pseudovirus neutralization titers (pVNT50) were calculated using the Reed-Muench method.


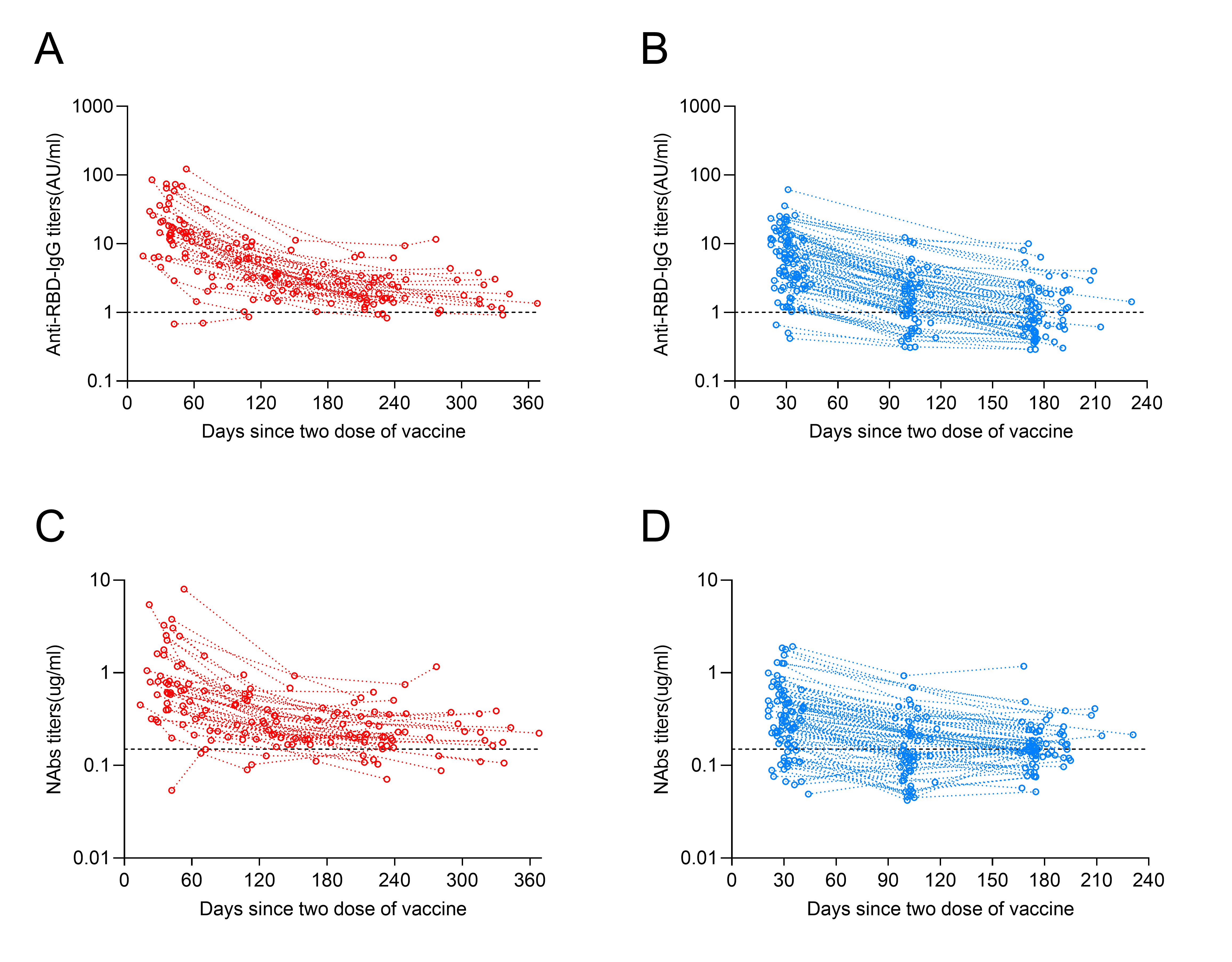


**Supplementary Figure 1. Antibody responses after primary inactivated vaccination in CHB children and adults with longitudinal blood samples.**

The kinetic changes in the titers of anti-RBD-IgG over time in CHB children (n=58) (**A)** and adults (n=91)(**B**). The kinetic changes in the titers of NAbs over time in CHB children(**C)** and adults(**D**). The dotted lines represent cut-off values. Anti-RBD-IgG, anti-receptor-binding-domain immunoglobulin G; NAbs, neutralizing antibodies.

**
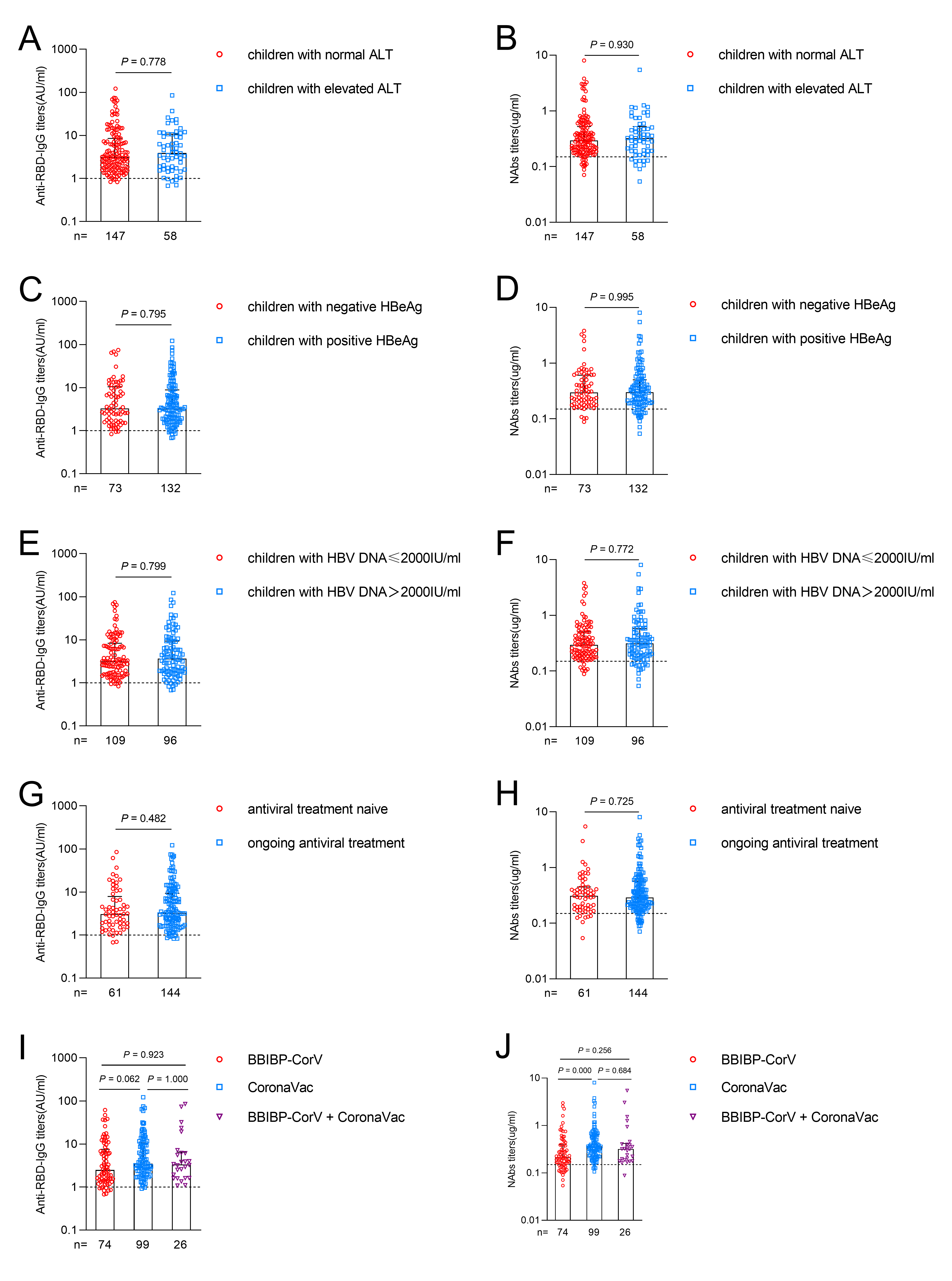
**

**Supplementary Figure 2. Antibody responses after primary inactivated vaccination in different subgroups of CHB children.** (**A, C, E, G, I**) Comparisons of the titers of anti-RBD IgG in CHB children grouped by ALT level (**A**), HBeAg status (**C**), HBV DNA level (**E**), antiviral situation (**G**), and vaccine type (**I**). (**B, D, F, H, J**) Comparisons of the titers of NAbs in CHB children grouped by ALT level (**B**), HBeAg status (**D**), HBV DNA level (**F**), antiviral situation (**H**), and vaccine type (**J**). The plots were shown as median values with the 95% confidence interval. The dotted lines represent cut-off values. anti-RBD IgG, anti-receptor-binding-domain immunoglobulin G; NAbs, neutralizing antibodies; ALT, alanine aminotransferase; HBeAg, hepatitis B e antigen. The Mann‒Whitney U test was used for comparisons between groups.


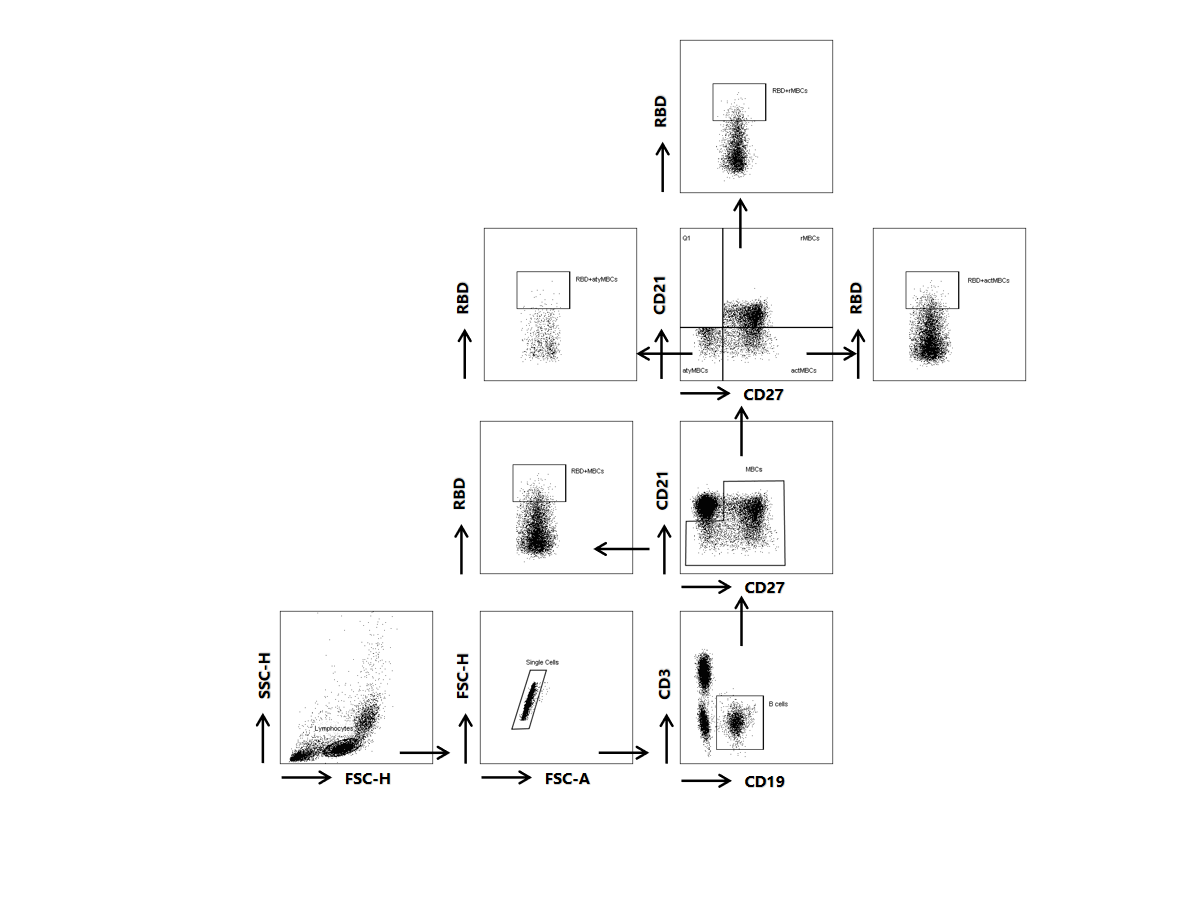


**Supplementary Figure 3. Gating strategy of flow cytometry for target cell population. RBD, receptor-binding domain.**

**Supplementary Table 1. The demographic and clinical characteristics of CHB adults**

| **Variables** | **CHB adults (n=351)** |
| --- | --- |
| Age (years) | 40(33-48) |
| Sex (male, n (%)) | 198(56.4%) |
| BMI (kg/m^2) | 22.86(20.98-24.84) |
| Vaccine type^#^ |  |
| BBIBP-CorV, n (%) | 141(40.2%) |
| CoronaVac, n (%) | 176(50.1%) |
| Mixed vaccination, n (%) | 32(9.1%) |
| WBC (10^9/L) | 5.34(4.66-6.28) |
| PLT (10^9/L) | 191(163-224) |
| ALT (U/L) | 22(17-35) |
| Nomal ALT, n (%) | 285(81.2%) |
| HBeAg negative, n (%) | 251(71.5%) |
| HBV DNA ≤2000 IU/ml, n (%) | 284(80.9%) |
| Antiviral treatment (Yes, n (%)) | 220(62.7%) |
| Days after two doses of vaccines (n=478) | 58(32-100) |
| Blood collection frequency |  |
| Multiple time point donors (two to three times) | 25.9% (91/351) |
| Single–time point donors | 74.1% (260/351) |

Abbreviations: CHB, chronic hepatitis B; IQR，interquartile range；BMI, body mass index; WBC, white blood cell; PLT, platelet; ALT, alanine aminotransferase; HBeAg, hepatitis B e antigen. ^#^ Vaccine types of 2 adults are unclear.

**Supplementary Table 2. Characteristics of participants included in the comparisons of neutralization against WT and Omicron subvariants.**

| **Variables** | **CHB children (n=15)** | **CHB adults (n=16)** | **P value** |
| --- | --- | --- | --- |
| Age (years) | 8.0(7.0-13.0) | 35.5(34.0-40.5) | 0.000 |
| Sex (male, n (%)) | 8 (53.3%) | 8 (50.0%) | 1.000 |
| Vaccine type |  |  | 0.677 |
| BBIBP-CorV, n (%) | 8 (53.3%) | 6 (37.5%) |  |
| CoronaVac, n (%) | 6 (40.0%) | 8 (50.0%) |  |
| Mixed vaccination, n (%) | 1 (6.7%) | 2 (13.3%) |  |
| Days after two doses of vaccines | 35.0(27.0-41.0) | 29.0(26.3-32.8) | 0.060 |

Abbreviations: CHB, chronic hepatitis B; IQR，interquartile range. The Mann‒Whitney U test was used for comparisons between groups. The chi-square test was used for categorical variables.

**Supplementary Table 3. Factors affecting antibody response to inactivated SARS-CoV-2 vaccines in CHB children**

|  | **anti-RBD-IgG** | | ***P* value** | **CoV-2 NAbs** | | ***P* value** |
| --- | --- | --- | --- | --- | --- | --- |
|  | Antibody low  (n=102) | Antibody high  (n=103) |  | Antibody low  (n=102) | Antibody high  (n=103) |  |
| Age (years) | 11(8-14) | 8(5-12) | 0.000 | 11(8-13) | 8(5-12) | 0.000 |
| Sex(boy, n (%)) | 66(64.7%) | 56(54.4%) | 0.132 | 69(67.6%) | 53(51.5%) | 0.018 |
| BMI (Kg/m^2^) | 16.90(14.50-19.95) | 15.34(14.44-17.45) | 0.161 | 16.90(14.38-20.00) | 15.34(14.45-17.32) | 0.069 |
| Vaccine type（BBIBP-CorV/CoronaVac/Mixed vaccination)# | 41/46/12 | 33/53/14 | 0.470 | 50/36/12 | 24/63/14 | 0.000 |
| Days after two doses of vaccine | 194(130-241) | 60(39-113) | 0.000 | 163(121-233) | 62(39-132) | 0.000 |
| ALT(U/L) | 24.0(17.0-39.0) | 27.5(21.0-48.0) | 0.052 | 28.0(18.0-46.0) | 26.0(20.0-46.8) | 0.150 |
| HBeAg status (positive, n (%)) | 66(64.7%) | 66(64.1%) | 0.925 | 65(63.7%) | 67(65.0%) | 0.843 |
| HBV DNA (≤2000 IU/ml/  ＞2000IU/ml) | 57/45 | 52/51 | 0.439 | 56/46 | 53/50 | 0.621 |
| Antiviral treatment (Yes, n (%)) | 70(68.6%) | 74(71.8%) | 0.614 | 74(72.5%) | 70(68.0%) | 0.473 |

Abbreviations: CHB, chronic hepatitis B; anti-RBD-IgG, spike receptor-binding domain IgG antibody; NAbs, neutralizing antibodies; BMI, body mass index; ALT, alanine aminotransferase; HBeAg, hepatitis B e antigen. ^#^ Vaccine types of 4 children are unclear. The Mann‒Whitney U test was used for comparisons between groups. The chi-square test was used for categorical variables.
